# Receipt of Selected Clinical Preventive Services by Adults — United States, 2011–2012

**Published:** 2015-07-17

**Authors:** Jared B. Fox, Frederic E. Shaw

**Affiliations:** 1 Office of Health System Collaboration, Office of the Associate Director for Policy, CDC; 2 Center for Surveillance, Epidemiology and Laboratory Services, CDC

Preventive services are available for nine of the ten leading causes of death in the United States (1). The Affordable Care Act (ACA) has reduced cost as a barrier to care by expanding access to insurance and requiring many health plans to cover certain recommended preventive services without copayments or deductibles (1). To establish a baseline for the receipt of these services for monitoring the effects of the law after 2012, CDC analyzed responses from persons aged ≥18 years in the National Health Interview Survey (NHIS) for the years 2011 and 2012 combined. NHIS is an in-person interview administered annually to a nationally representative sample of the noninstitutionalized, U.S. civilian population. This report summarizes the findings for nine preventive services covered by the ACA. Having health insurance or a higher income was associated with higher rates of receiving these services, affirming findings of previous studies (2). Securing health insurance coverage might be an important way to increase receipt of clinical preventive services, but insurance coverage is not sufficient to ensure that everyone is offered or uses clinical services proven to prevent disease. Greater awareness of ACA provisions among the public, public health professionals, partners, and health care providers might help increase the receipt of recommended services (3).

The responses to questions about the receipt of nine clinical preventive services recommended by the U.S. Preventive Services Task Force (USPSTF) or the Advisory Committee on Immunization Practices (ACIP) were analyzed to identify receipt rates for the clinical services (Table 1). The nine preventive services are among dozens of services for adults covered with no copayments or deductibles under certain health plans according to the ACA*†: 1)blood pressure screening, 2) cholesterol screening, 3) colon cancer screening, 4) diet counseling, 5) fasting blood glucose test (diabetes screening), 6) hepatitis A vaccination, 7) hepatitis B vaccination, 8) mammogram (breast cancer screening), and 9) Papanicolaou (Pap) test (cervical cancer screening). While clinical guidelines change over time (i.e., adjusting the recommended periodicity or risk factors for which the service is indicated), it is important to consistently monitor receipt rates for the underlying clinical services for accurate year-to-year comparisons. Asked annually since 2011, the NHIS survey questions used for this analysis are designed to consistently measure receipt of the services each survey year and to improve accuracy of responses by limiting recall of service receipt to 12 months where possible; for hepatitis A and B vaccinations, respondents were asked if they had ever received this service (Table 1). Only 15 preventive services (these nine services and six others previously reported on in 2014 [4]) are included in both the ACA’s coverage requirements and the annual NHIS.

To increase sample sizes and improve the reliability of estimates for this analysis, NHIS data from the sample adult core questionnaires in 2011 and 2012 were combined. From within each family in each household identified, one adult (aged ≥18 years) was randomly selected to complete the questionnaire.§ NHIS 2011 and 2012 adult core samples included 33,014 and 34,525 respondents, respectively, and the overall response rates were 66.3% and 61.2%.

Participants were asked whether they had health insurance at the time of the interview. They were considered uninsured if they reported currently not having private health insurance, Medicare, Medicaid, Children’s Health Insurance Program, a state-sponsored or other government-sponsored health plan, or a military plan, or if they had only a private plan that paid for one type of service (e.g., injury or dental care) or had only Indian Health Service coverage.¶ Multiple imputations were performed on family income to account for missing responses to income questions.** NHIS data were adjusted for nonresponse and weighted to provide national estimates of insurance status and receipt of preventive care; 95% confidence intervals were calculated that took into account the survey’s multistage probability sample design. Generalized linear modeling and t-tests were used to calculate prevalence ratios and determine statistical significances of differences in receipt of preventive services between persons in three categories: 1) insured versus uninsured, 2) current family incomes >200% of the federal poverty level (FPL)†† versus current family incomes ≤200% of the FPL, and 3) any private health insurance versus only public coverage. Analysis for each service was restricted to persons of the age and sex for who receipt of that service is recommended (Table 1).

For the nine services examined, prevalence of receipt of service in the queried timeframe was as follows: hepatitis A vaccination, 12.7%; colon cancer screening, 23.6%; diet counseling, 26.9%; hepatitis B vaccination, 38.8%; diabetes screening, 45.3%; cervical cancer screening, 59.4%; breast cancer screening, 61.6%; cholesterol screening, 70.0%; and blood pressure screening, 82.9% (Table 2). A statistically significant higher percentage of adults with health insurance received each of nine clinical preventive services compared with those who were uninsured (Table 2). Among the nine services, the service receipt prevalence ratio for those with insurance compared with those without insurance ranged from 1.39 for hepatitis B vaccination to 3.13 for colon cancer screening (Table 2).

Persons with family incomes >200% of the FPL received clinical preventive services at a statistically significant higher prevalence compared with those with incomes below that threshold for eight of nine services (all but hepatitis A vaccination) (Table 3). Among those eight services, the service receipt prevalence ratio for those with family incomes >200% of the FPL compared with those with incomes ≤200% of the FPL ranged from 1.06 for hepatitis B vaccination to 1.43 for breast cancer screening (Table 3).

Persons with private health insurance received preventive services at a statistically significant higher prevalence for two of nine services, and at a lower prevalence for four of nine services, compared with those with only public insurance (Table 4).

## Discussion

During 2011–2012, those with insurance or with higher incomes were more likely than those without coverage or with lower incomes, respectively, to have received nine preventive services during the identified time period. This supports previously published studies, including one that found prevalence ratios in the range of 1–3 for those with insurance receiving preventive services in the prior year compared with those without coverage (2,4).

This report could serve as a baseline for tracking the effects of some of the ACA’s preventive care provisions that might occur after 2012. Since the ACA began to require certain plans to cover clinical preventive services as early as September 2010, the data from the 2011–2012 study period might include some of the early impact of the law. Any early impact included might be limited for several reasons: 1) a high number of persons remained uninsured during 2011–2012; 2) there was little awareness of the preventive care provisions of the new law; and 3) many plans were not yet subject to the preventive services provisions because of grandfathering and other factors (1,5–7). Monitoring the trend of service receipt rates over time could provide insight into how the service receipt gaps relating to income and insurance status might change as more persons gain coverage that includes the ACA’s preventive service coverage requirements.

The findings in this report are subject to at least six limitations. First, receipt of preventive services was self-reported and might be subject to recall bias, particularly for lifetime receipt of services like vaccinations that are routinely administered to young children rather than adults. Second, inferences from these results are limited by differences in time between when the questions were asked and when the services were received. For example, NHIS identifies whether the respondent is insured at the time of interview; however, depending on the service, NHIS asks whether the respondent received preventive care in the last 12 months, or ever during their lifetime. Currently uninsured respondents might have received preventive care during a time when they had insurance, or vice versa. Third, some of the services might have been received as diagnostic measures instead of for prevention. Fourth, the results of this analysis identify the rates of service receipt during the 12 months before interview, or ever in life, but cannot be seen as measures of adherence to guidelines because of differences between the annual survey questions and the official recommendation for these nine services. Fifth, this cross-sectional analysis does not demonstrate causation and does not include other possible confounders that might be associated with service receipt rates. For example, those with higher incomes might also be more likely to have health insurance, and vice versa. Finally, NHIS is limited to noninstitutionalized civilians, excluding certain populations (e.g., the institutionalized and the military) that might be especially likely to receive recommended preventive services.

**Summary**

What is already known on this topic?

Rates of receipt of some clinical preventive services by adults are higher for persons with insurance coverage or higher incomes. The Affordable Care Act’s expansions of health insurance access and coverage requirements for clinical preventive services were developed to increase access to health services to improve the health of the population.

What is added by this report?

Analysis of combined adult responses to the National Health Interview Survey in 2011 and 2012 indicated that persons with health insurance were more likely to have received preventive services than persons without insurance for each of nine services. Further, persons with higher income were more likely to have received preventive services than persons with lower income for eight of nine services.

What are the implications for public health practice?

Increased insurance coverage could lead to a significant increase in receipt of preventive care and improvements in population health.

All new private health plans, alternative benefit plans for the newly Medicaid eligible, and Medicare now provide coverage with no copayments or deductibles for many recommended clinical preventive services as part of the ACA (1). These provisions might have the greatest impact for higher-cost services like certain colorectal cancer screening methods. Of the nine services examined, colon cancer screening had the highest service receipt prevalence ratio, 3.13, for those with insurance compared with those without insurance. While insurance coverage is not the only barrier to receiving services, efforts to increase enrollment and coverage retention could help increase receipt of preventive services and reduce avoidable complications from illness, long-term health care costs, and premature deaths (8).

## Figures and Tables

**TABLE 1:** Comparison of recommendations from the United States Preventive Services Task Force (USPSTF) and the Advisory Committee on Immunization Practices (ACIP) with questions regarding nine recommended clinical preventive services in the National Health Interview Survey (NHIS)—United States, 2011–2012

Clinical preventive service (age group [yrs])	Recommendation	Question to NHIS participants	Key distinctions for this analysis of use of recommended services
Blood pressure screening (≥18)	Screening for high blood pressure is recommended for adults aged ≥18 years. The optimal screening interval is uncertain, but a one- or two-year screening interval, depending on risk factors, is one example highlighted by the USPSTF.*	“During the past 12 months, have you had your blood pressure checked by a doctor, nurse, or other health professional?” Response analyzed for persons aged ≥18 years.	There is no specific recommended screening interval, which differs from the survey question timeframe (12 months). The results of this analysis identify service use and cannot determine adherence to guideline.
Breast cancer screening (women, 50–74)	Screening via mammography every two years is recommended for all women aged 50–74 years.*†	“Have you had a mammogram during the past 12 months?” Response analyzed for women aged 50–74 years.	The recommended screening interval (2 years) differs from the survey question timeframe (12 months). The results of this analysis identify service use and cannot determine adherence to guideline.
Cervical cancer screening (women, 21–65)	Screening via cytology (Pap test) is recommended every three years for women aged 21–65 years. Women aged 30–65 years can be screened every 5 years by adding a human papillomavirus test to the cytology.*§	“Have you had a Pap smear or Pap test During the past 12 months?” Response analyzed for women aged 21–65 years.	The recommended screening interval (three or five years) for cytology differs from the survey question timeframe (12 months). The results of this analysis identify service use and cannot determine adherence to guideline.
Cholesterol screening (men, ≥35)	Screening for lipid disorders via a cholesterol test is recommended for all men aged ≥35 years.¶ The optimal screening interval is uncertain, but the USPSTF states that 5 years is an example of a reasonable interval.*	“During the past 12 months, have you had your blood cholesterol checked by a doctor, nurse, or other health professional?” Response analyzed for men aged ≥35 years.	There is no specific recommended screening interval, which differs from the survey question timeframe (12 months). The results of this analysis identify service use and cannot determine adherence to guideline.
Colon cancer screening (50–75)	Colorectal cancer screening is recommended for all adults aged 50–75 years. Recommended screening interval varies by screening method: 1 year for high-sensitivity fecal occult blood testing (FOBT); five years for sigmoidoscopy with FOBT every 3 years; 10 years for colonoscopy.*	“During the past 12 months, have you had any test done for colon cancer?” Response analyzed for persons aged 50–75 years.	The recommended screening interval (1–10 years) differs from the survey question timeframe (12 months). The results of this analysis identify service use and cannot determine adherence to guideline.
Diabetes screening (≥18)	Screening for type 2 diabetes is recommended for asymptomatic adults with sustained blood pressure greater than 135/80 mmHg. The optimal screening interval is uncertain, but the American Diabetes Association recommends a 3-year interval.*	“Have you had a fasting test for high blood sugar or diabetes during the past 12 months?” Response analyzed for persons aged ≥18 years.	The recommended screening interval is uncertain and the suggested interval (3 years) differs from the survey question timeframe (12 months). Also, fasting blood glucose is just one of three methods recommended for diabetes screening. Further, this analysis identifies the screening rate for all adults and not just those with sustained hypertension. The results of this analysis identify service use and cannot determine adherence to guideline.
Diet counseling (≥18)	Intensive behavioral dietary counseling is recommended for adults with known risk factors for cardiovascular and diet-related chronic disease. The optimal screening and counseling interval is not known.*	“During the past 12 months, has a doctor or other health professional talked to you about your diet?” Response analyzed for persons aged ≥18 years.	The recommended counseling interval is uncertain and differs from the survey question timeframe (12 months). Further, this analysis identifies the counseling rate for all adults and not just those with specific risk factors. Additionally, the survey does not clarify whether the conversation with the health professional met the standard of “intensive behavioral counseling” called for in the recommendation. The results of this analysis identify service use and cannot determine adherence to guideline.
Hepatitis A vaccination (19–49)	Hepatitis A vaccination recommendations are universal for children aged 1 year. The recommendations for adults are limited to high-risk persons and “anyone seeking immunization.”**	“How many hepatitis A shots did you receive?”†† (response of greater than two is coded as fully vaccinated)** Response analyzed for persons aged ≥18 years.	The recommendations for adults include those aged ≥19 years. This analysis focuses on those aged 19–49 years for consistency with other CDC reports of hepatitis A vaccination rates among adults.§§ Further, this analysis identifies the vaccination rate for all adults and not just those with specific risk factors. The results of this analysis identify service use and cannot determine adherence to guideline.
Hepatitis B vaccination (19–49)	Hepatitis B vaccination recommendations are universal for children. The recommendations for adults include high-risk persons and “anyone seeking immunization.”**	“Did you receive at least three doses of the hepatitis B vaccine, or greater than three doses?”*†† (response of three or greater is coded as fully vaccinated)** Response analyzed for persons aged ≥18 years.	The recommendations for adults include those aged ≥19 years. This analysis focuses on those aged 19–49 years for consistency with other CDC reports of hepatitis B vaccination rates among adults.§§ Further, this analysis identifies the vaccination rate for all adults and not just those with specific risk factors. The results of this analysis identify service use and cannot determine adherence to guideline.

**Abbreviation:** Pap test = Papanicolaou test.

* Source: USPSTF.

† While the USPSTF currently recommends biennial mammography for women aged 50–74 years, the ACA coverage requirement includes women aged 40–74.

§ The current USPSTF recommendations for cervical cancer screening were released in March 2012, after much of the data for this study were collected. Prior to the 2012 update, the USPSTF recommended only triennial screening via Pap test.

¶ Four groups of persons are recommended for cholesterol screening at grade A and B: 1) men aged ≥35 years; 2) men aged 20–35 years at increased risk for coronary heart disease; 3) women aged ≥45 years at increased risk for coronary heart disease; and 4) women aged 20–45 years at increased risk for coronary heart disease. This report only includes data for men aged ≥35 years because it was not possible to estimate increased risk for coronary heart disease in the study population.

** Source: ACIP.

†† Includes services received at any age.

§§ Williams WW, Lu PJ, O’Halloran A, et al. Noninfluenza vaccination coverage among adults—United States, 2012. MMWR Morb Mortal Wkly Rep 2014;63:95–102.

**TABLE 2:** Percentage of adults in the recommended populations who received nine clinical preventive services, by health insurance status — National Health Interview Survey, United States, 2011–2012

Clinical preventive service (age group [yrs])	Insured			Uninsured			Prevalence ratio, insured/uninsured*		Total	
			
	No.	Weighted %	(95% CI)	No.	Weighted %	(95% CI)	Ratio†	(95% CI)	Weighted %	(95% CI)
Blood pressure screening§ (≥18)	54,265	87.9	(87.6–88.3)	11,873	56.3	(55.2–57.5)	1.56	(1.53–1.59)	**82.9**	**(82.5–83.3)**
Breast cancer screening§ (women, 50–74)	11,827	65.4	(64.3–66.4)	1,478	26.4	(23.8–28.9)	2.48	(2.25–2.73)	**61.6**	**(60.5–62.6)**
Cervical cancer screening§ (21–65)	21,932	64.2	(63.4–65.0)	5,649	38.1	(36.6–39.6)	1.68	(1.62–1.75)	**59.4**	**(58.7–60.2)**
Cholesterol screening§ (men, ≥35)	17,704	76.5	(75.7–77.2)	3,330	31.3	(29.5–33.2)	2.44	(2.30–2.59)	**70.0**	**(69.2–70.8)**
Colon cancer screening§ (50–75)	21,958	25.4	(24.7–26.0)	2,844	8.1	(7.0–9.3)	3.13	(2.71–3.61)	**23.6**	**(23.0–24.2)**
Diabetes screening§ (≥18)	53,725	49.9	(49.2–50.6)	11,813	21.4	(20.6–22.2)	2.33	(2.25–2.43)	**45.3**	**(44.7–45.9)**
Diet counseling§ (≥18)	54,210	29.2	(28.7–29.7)	11,875	14.9	(14.2–15.6)	1.97	(1.87–2.06)	**26.9**	**(26.5–27.3)**
Hepatitis A vaccination, full¶ (19–49)	21,883	13.8	(13.1–14.5)	7,746	9.2	(8.3–10.1)	1.49	(1.35–1.65)	**12.7**	**(12.1–13.3)**
Hepatitis B vaccination, full¶ (19–49)	24,046	41.5	(40.6–42.4)	8,367	29.8	(28.5–31.2)	1.39	(1.32–1.46)	**38.8**	**(38.0–39.5)**

**Abbreviation:** CI = confidence interval.

* Generalized linear modeling was used to identify statistical significance of differences between insured and uninsured persons receiving service.

† p<0.001.

§ Service received within preceding 12 months.

¶ Ever received service.

**TABLE 3:** Percentage of adults in the recommended populations who received nine clinical preventive services, by family income level — National Health Interview Survey, United States, 2011–2012

Clinical preventive service (age [yrs])	Income >200% FPL			Income ≤200% FPL			Prevalence ratio, higher income/lower income*	
		
	No.	Weighted %	(95% CI)	No.	Weighted %	(95% CI)	Ratio	(95% CI)
Blood pressure screening† (≥18)	40,120	86.2	(85.8–86.6)	26,221	76.9	(76.2–77.7)	1.12§	(1.11–1.13)
Breast cancer screening† (women, 50–74)	8,749	67.8	(66.6–69.0)	4,588	47.3	(45.5–49.1)	1.43§	(1.37–1.49)
Cervical cancer screening† (21–65)	16,316	64.4	(63.5–65.3)	11,339	50.9	(49.7–52.0)	1.27§	(1.23–1.30)
Cholesterol screening† (men, ≥35)	14,489	73.6	(72.7–74.5)	6,592	60.6	(59.2–62.0)	1.22§	(1.18–1.25)
Colon cancer screening† (50–75)	16,779	25.1	(24.4–25.8)	8,079	19.8	(18.8–20.9)	1.26§	(1.19–1.34)
Diabetes screening† (≥18)	39,764	48.7	(48.0–49.4)	25,975	39.2	(38.2–40.1)	1.24§	(1.21–1.28)
Diet counseling† (≥18)	40,081	28.2	(27.7–28.7)	26,205	24.7	(24.0–25.3)	1.14§	(1.11–1.18)
Hepatitis A vaccination, full¶ (19–49)	17,023	13.0	(12.3–13.6)	12,703	12.3	(11.3–13.2)	1.06**	(0.97–1.15)
Hepatitis B vaccination, full¶ (19–49)	18,525	39.7	(38.8–40.5)	14,006	37.4	(36.1–38.8)	1.06††	(1.02–1.10)

**Abbreviations:** CI = confidence interval; FPL = federal poverty level.

* Generalized linear modeling was used to identify statistical significance of differences between persons at higher income level and lower income level receiving service.

† Service received within preceding 12 months.

§ p<0.001.

¶ Ever received service.

** p>0.05.

†† p<0.01.

**TABLE 4:** Percentage of adults in the recommended populations who received nine clinical preventive services, by source of health insurance coverage — National Health Interview Survey, United States, 2011–2012

Clinical preventive service (age group [yrs])	Private insurance receiving service			Only public insurance receiving service			Prevalence ratio, private/public*	
		
	No.	Weighted %	(95% CI)	No.	Weighted %	(95% CI)	Ratio	(95% CI)
Blood pressure screening† (≥18)	38,462	87.2	(86.8–87.6)	15,794	90.0	(89.5–90.6)	0.97§	(0.96–0.98)
Breast cancer screening† (women, 50–74)	8,044	68.6	(67.3–69.9)	3,781	57.6	(55.8–59.4)	1.19§	(1.15–1.23)
Cervical cancer screening† (21–65)	16,511	65.8	(64.9–66.6)	5,421	58.3	(56.6–60.1)	1.13§	(1.09–1.16)
Cholesterol screening† (men, ≥35)	12,445	74.6	(73.7–75.6)	5,255	81.4	(80.0–82.7)	0.92§	(0.90–0.94)
Colon cancer screening† (50–75)	14,734	25.0	(24.2–25.8)	7,221	26.3	(25.1–27.5)	0.95¶	(0.90–1.00)
Diabetes screening† (≥18)	38,114	47.6	(46.8–48.4)	15,602	56.3	(55.3–57.3)	0.85§	(0.83–0.87)
Diet counseling† (≥18)	38,426	28.0	(27.5–28.5)	15,774	32.6	(31.6–33.6)	0.86§	(0.83–0.89)
Hepatitis A vaccination, full** (19–49)	17,288	13.8	(13.0–14.5)	4,595	13.9	(12.6–15.1)	0.99¶	(0.90–1.09)
Hepatitis B vaccination, full** (19–49)	18,976	41.8	(40.8–42.8)	5,070	40.3	(38.7–41.8)	1.04¶	(0.99–1.08)

**Abbreviation:** CI = confidence interval.

* Generalized linear modeling was used to identify statistical significance of differences between persons with private insurance and only public insurance receiving service.

† Service received within preceding 12 months.

§ p<0.001.

¶ p>0.05.

** Ever received service.

## Footnotes

* For adults, the Affordable Care Act recognizes the U.S. Preventive Services Task Force (http://www.uspreventiveservicestaskforce.org) and the Advisory Committee on Immunization Practices (http://www.cdc.gov/vaccines/acip), as two of the organizations whose clinical preventive service recommendations receive coverage without copayments and deductibles for certain health plans.

† An interactive tool, available at http://www.cdc.gov/prevention, lets users identify which preventive services are recommended for a particular person and covered by the ACA.

§ Additional information available at http://www.cdc.gov/nchs/nhis/quest_data_related_1997_forward.htm.

¶ Consistent with other population surveys conducted by U.S. federal agencies, CDC does not regard Indian Health Service coverage as health insurance for the purpose of identifying uninsured populations.

** Additional information available at http://www.cdc.gov/nchs/nhis/2011imputedincome.htm and http://www.cdc.gov/nchs/nhis/2012imputedincome.htm.

†† In 2012, the FPL for a family of four was $46,100; additional information available at http://aspe.hhs.gov/poverty/12poverty.shtml and http://www.census.gov/hhes/www/poverty/about/overview/measure.html.
